# Multivendor fully automatic uncertainty management approaches for the intuitive representation of DME fluid accumulations in OCT images

**DOI:** 10.1007/s11517-022-02765-z

**Published:** 2023-01-24

**Authors:** Plácido Vidal, Joaquim de Moura, Jorge Novo, Marcos Ortega

**Affiliations:** 1 Centro de investigación CITIC, Universidade da Coruña, Campus de Elviña, s/n, A Coruña, 15071 Galicia Spain; 2 Grupo VARPA, Instituto de Investigación Biomédica de A Coruña (INIBIC), Universidade da Coruña, Xubias de Arriba, 84, A Coruña, 15006 Galicia Spain

**Keywords:** Optical coherence tomography, Diabetic macular edema, Confidence map generation, Transfer learning, Computer-aided diagnosis

## Abstract

Diabetes represents one of the main causes of blindness in developed countries, caused by fluid accumulations in the retinal layers. The clinical literature defines the different types of diabetic macular edema (DME) as cystoid macular edema (CME), diffuse retinal thickening (DRT), and serous retinal detachment (SRD), each with its own clinical relevance. These fluid accumulations do not present defined borders that facilitate segmentational approaches (specially the DRT type, usually not taken into account by the state of the art for this reason) so a diffuse paradigm is used for its detection and visualization. In this paper, we propose three novel approaches for the representation and characterization of these types of DME. A baseline proposal, using a convolutional neural network as backbone, another based on transfer learning from a general domain, and a third approach exploiting information of regions without a defined label. Overall, our baseline proposal obtained an AUC of 0.9583 ± 0.0093, the approach pretrained with a general-domain dataset an AUC of 0.9603 ± 0.0087, and the approach pretrained in the domain taking advantage of uncertainty, an AUC of 0.9619 ± 0.0073.

**Supplementary Information:**

The online version contains supplementary material available at 10.1007/s11517-022-02765-z.

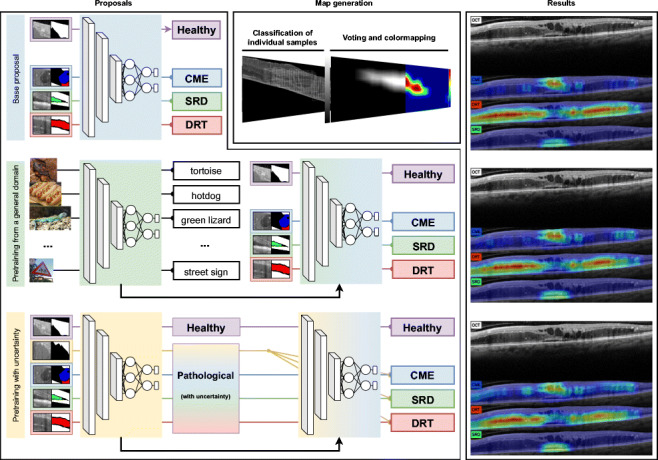

## Introduction

Due to modern consumption habits, there has been an increase in prevalence of diabetes where, if all the cases in the world were a separate country, would be the third largest in the world (and almost 1% of its population would die each year). Moreover, 79% of these cases live in developed countries, being expected to increase to 84% by 2045 [1]. The relevance of the study of this pathology lies not only in its wide prevalence, but also in its long-term consequences for the quality of life of those affected by it, as this pathology represents one of the main causes of blindness in developed countries [2]. This blindness is due to the deterioration of the delicate vascular network of the retina. These vascular structures begin to leak fluid into it, destroying its layered morphology. This type of diabetic retinopathy is called diabetic macular edema or DME [3].

**Fig. 1:**
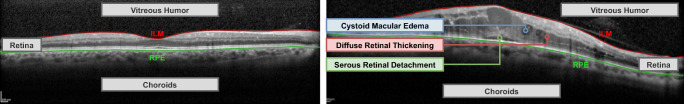
OCT images from a healthy retina (left) and a retina presenting the three studied types of DME (right). The limiting membranes of the retina are also indicated (Inner Limiting Membrane, ILM and Retinal Pigmented Epithelium, RPE)

Currently, in the reference clinical literature, these fluid accumulations are classified into three main types according to their textural features, morphology, and arrangement: cystoid macular edema (CME), serous retinal detachment (SRD), and diffuse retinal thickening (DRT). These patterns are based on features studied in optical coherence tomography (OCT) images [4–7], as they allow for a non-invasive cross-sectional representation of the retinal structures. In Fig. 1, we show an example of two OCT images: one without any fluid accumulation and other that presents all the three fluid types. Each of these types represents a different level of severity, as well as different complications for its treatment and diagnosis. The DRT type, as its name implies, represents a diffuse spongiform accumulation of fluid in the retina. This fluid accumulation is easier to treat, as the retinal tissues suffer minor lesions compared to the other two. However, it is the hardest to diagnose as it does not have defined limits and can present itself with texture and gray levels similar to normal retinal tissues. Additionally, this type of DME usually precedes the appearance of the other two, being an early indicator of the disease (thus, its identification and characterization are critical for an early diagnosis and correct recovery of the afflicted). On the other hand, the CME type presents fluid accumulations with cellular barriers and hiporreflective fluid accumulations (usually with similar patterns as the vitreous humor). Its the easiest to study, albeit in smaller sizes (called microcysts), its features and consequences vary in significance and are studied as a separated scenario in the clinical literature [8]. Finally, the SRD fluid accumulations usually represent the most critical type, affecting the central vision in the outermost layers (near the fovea and photoreceptors). These last two fluid accumulations are the hardest to treat, as they can completely deform the retinal structures and even leave scar tissues when reabsorbed. Depending on the degree of affliction (or if left untreated), the treatments can range from pharmacologic to invasive surgical procedures [9].

### Related works

The need of procedures that allow for a robust and repeatable monitoring of these fluid accumulations resulted in the proposal of different computer-aided methodologies for its diagnosis. Originally, the prevalent paradigm was based on obtaining a defined segmentation of general fluid accumulations. For this strategy, methodologies based on classical learning [10–14] were proposed, based on both shape and texture constraints. On the other hand, more recent proposals [15–20] base its segmentation on variants of the U-Net architecture [21]: an encoder/decoder with skip connections between them (a common strategy in the medical imaging domain). Additionally, these works focus only on, at most, the segmentation of the fluid accumulations depending on their location in the retinal layers (subretinal fluid, intraretinal fluid, and a pigment epithelial detachment). Due to the difficulty of identification of diffuse fluid accumulations (such as in the extreme case of the DRT), only a limited number of works have considered it [22–24].

However, as stated, these fluid accumulations often present diffuse limits that cannot be segmented (even more so regarding the aforementioned DRT type). For this reason, an alternative paradigm was proposed to study these DME fluid types. What originally started on the classification of independent windows of a given size and a library of texture and intensity features [25, 26], it developed into a way to generate diffuse representations of the model confidence by means of a voting strategy [27–29] (albeit only focused on cystoid fluid accumulations). This paradigm showed promising results with the three aforementioned reference types of fluid accumulations [30], albeit presenting some limitations due to its backbone based on classical learning approaches which rely on a predefined feature library [31].

### Contributions

In this paper, we propose three novel approaches to address the challenge of DME fluid characterization with the aforementioned diffuse paradigm. First, as a baseline proposal, we introduce the use of deep convolutional networks as the classifier backbone. This network is trained to classify the different independent samples that are being used to generate the confidence map of the respective fluid accumulations. By using these deep learning networks, it is possible to completely eliminate the need for a library of features, creating ad hoc ones for each particular scenario.

As we previously discussed, these fluid accumulations present regions where no accurate labeling is possible. We refer to them as “regions with associated uncertainty,” since we do not have a clear classification of the specific fluid type to which they belong, merely their pathological nature. Our first proposal does not take these regions into consideration, forcing an inference of their label without any prior knowledge.

To overcome this inherent uncertainty, we propose two alternatives. First, a methodology based on a pretraining from a generalist domain. Specifically, using the ImageNet dataset [32, 33]. Subsequently, we perform a knowledge transfer to our specific domain, in order to adapt the filters that are learned in said prior training. Thus, the associated uncertainty of our domain is compensated by the features learned in a richer domain, allowing to elicit information not considered by the initial model in order to determine the most probable class in these regions with uncertainty.

However, the exploitation and adaptation of features from a generalist domain do not necessarily account for all the information gaps associated to the diffuse domain. For this reason, we propose a third approach to estimate the patterns associated with these weakly labeled regions. Instead of conducting a pretraining on a generalist domain, we first train the model for a binary classification, where samples are either pathologic or non-pathologic. However, within the pathological class, we include patterns from regions with associated uncertainty (which, as we noted above, present pathological patterns, but we do not know their associated DME subtype). Then, we replace the head of the convolutional network to classify the DME subtypes and resume the training, this time without considering the samples associated with uncertainty (since we do not have information for these regions at these levels of granularity). This way, we obtain a model capable of classifying the fluid subtypes, but that has already developed filters during the pretraining stage to take into consideration these regions with associated uncertainty. Moreover, not only this last approach allows the model to explicitly consider these regions, but also requiring significantly less resources compared to the approximation exploiting a generalist domain.

Thus, we propose these three novel approaches for the characterization and representation of different types of DME. Additionally, we evaluate these proposals on two of the main OCT devices in the clinical domain, where we test wether these approaches are able to obtain robust results even in regions where segmentation-based approaches are not able to obtain explicit results.

This paper is organized as follows: in Section 2 (Dataset and resources), we list and describe all the resources needed to perform this work (both dataset and software resources); Section 3 (Methodology) presents the steps followed to develop this work, the characteristics of all the stages that are involved, and the precise training configuration; Section 4 (Results and discussion) presents the metrics resulting from the conducted experiments, example images of the commented scenarios, and a discussion on the significance of each case. Finally, Section 5 (Conclusions) includes a final summary and commentary on the presented work, as well as future lines of research.

## Dataset and resources

For this work, to study the capabilities of our proposal to integrate patterns from multiple devices, a multivendor dataset composed by 356 OCT images was used. These images were taken with two representative OCT devices of the field: a CIRRUS^TM^ HD-OCT 500 Carl Zeiss Meditec and a HRA+OCT SPECTRALIS®; from Heidelberg Engineering, Inc. From the CIRRUS device, a total of 177 images were considered, while 179 from the Spectralis device. The OCT images were captured from both left and right eyes with different device configurations and ranging in resolution from 714 × 291 pixels to 1535 × 496 pixels in the Spectralis dataset and 682 × 446 pixels to 1680 × 1050 pixels in the Cirrus dataset. These images contain both images from patients afflicted by different severity levels by DME and healthy ones. The protocol to obtain these images from live clinical practice and its study was conducted in accordance with the Declaration of Helsinki and approved by the Ethics Committee of Investigation from A Coruña/Ferrol.

**Fig. 2:**
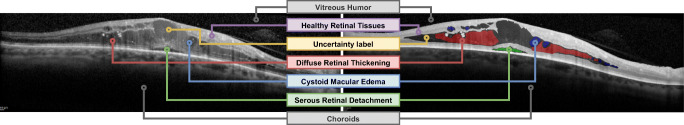
Examples of labeled regions in an OCT image and the label mask established by the experts

The dataset was labeled by two experts in the domain. In order for the system and derived metrics to take into consideration the inherent heterogeneity associated with the subjectivity of a human expert (critical especially in the domain of this work with its associated uncertainty), both experts labeled a random half of the dataset. Nonetheless, the proportion of images from each device was preserved in each of the labeled subsets. Both experts agreed beforehand the labeling protocol and standard to be followed, as well as a consensus on what defines the different considered classes. This protocol states that each class was only marked as positive if the expert had absolute certainty that the pixels that the mask overlaps belong to the given class. Otherwise, the label is established as uncertain. Thus, in Fig. 2, we can see an example of this labeling.

As software resources, for training and downloading all the models, we used the PyTorch library version 1.9.0+cu111 and Torchvision 0.10.0+cu111. For the calculation of metrics and data processing, we have used Scikit-Learn version 0.23.1 and NumPy version 1.19.5. Finally, for the generation of the maps, it was necessary to use the OpenCV-Python library version 4.1.2 and SciPy 1.5.2. All the previously mentioned experiments and libraries were executed in Python version 3.6.9 (default, Jan 26 2021). A model trained in ImageNet was used for the knowledge transfer approach from an external domain [32, 33].

### Dataset creation

**Fig. 3:**
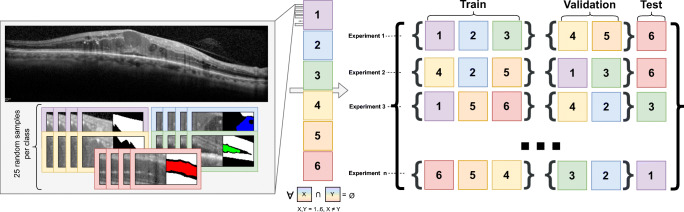
Diagram of the strategy followed to extract and distribute the samples in the dataset during experimentation

In Fig. 3, we present a diagram that represents the process of creating the dataset. First, we performed the sample extraction for each available image (as the map generation strategy uses windows of this size to generate the final result). Taking the labeled masks as reference, we randomly collect 25 samples for each label present in the image: healthy, CME, DRT, SRD, and region of uncertainty (the latter including only inner retinal regions, no sample is obtained from the vitreous humor or choroid except for the borderline regions of the retina). Each of these samples are obtained from a 64px × 64px window from the retinal regions, as performance degrades on larger or smaller window sizes [26, 34]. In the event that a sample partially falls outside the image, we mirror the edge patterns to complete the missing information. After the this process, we obtain a total of 8900 samples centered in healthy regions, 7286 samples centered in CME regions, 6241 samples centered in DRT regions, 1975 samples centered in SRD regions, and 8900 samples centered in regions with associated uncertainty.

Once we have created the sample library, we divide the dataset into six folds at image level, so no data leakages between training, validation, and test sets are possible. Subsequently, to perform the cross-validation, we explore all the combinations between these folds into training, validation, and test. In particular, we select three folds for training, two for validation, and one for test in each iteration. This cross-validation allows for the final metrics to be more robust in case the dataset presents any unbalance, as every possible combination of the folds is contemplated. This way, to explore all the possible fold combinations, we perform a total of 60 experiments.

## Methodology

**Fig. 4:**
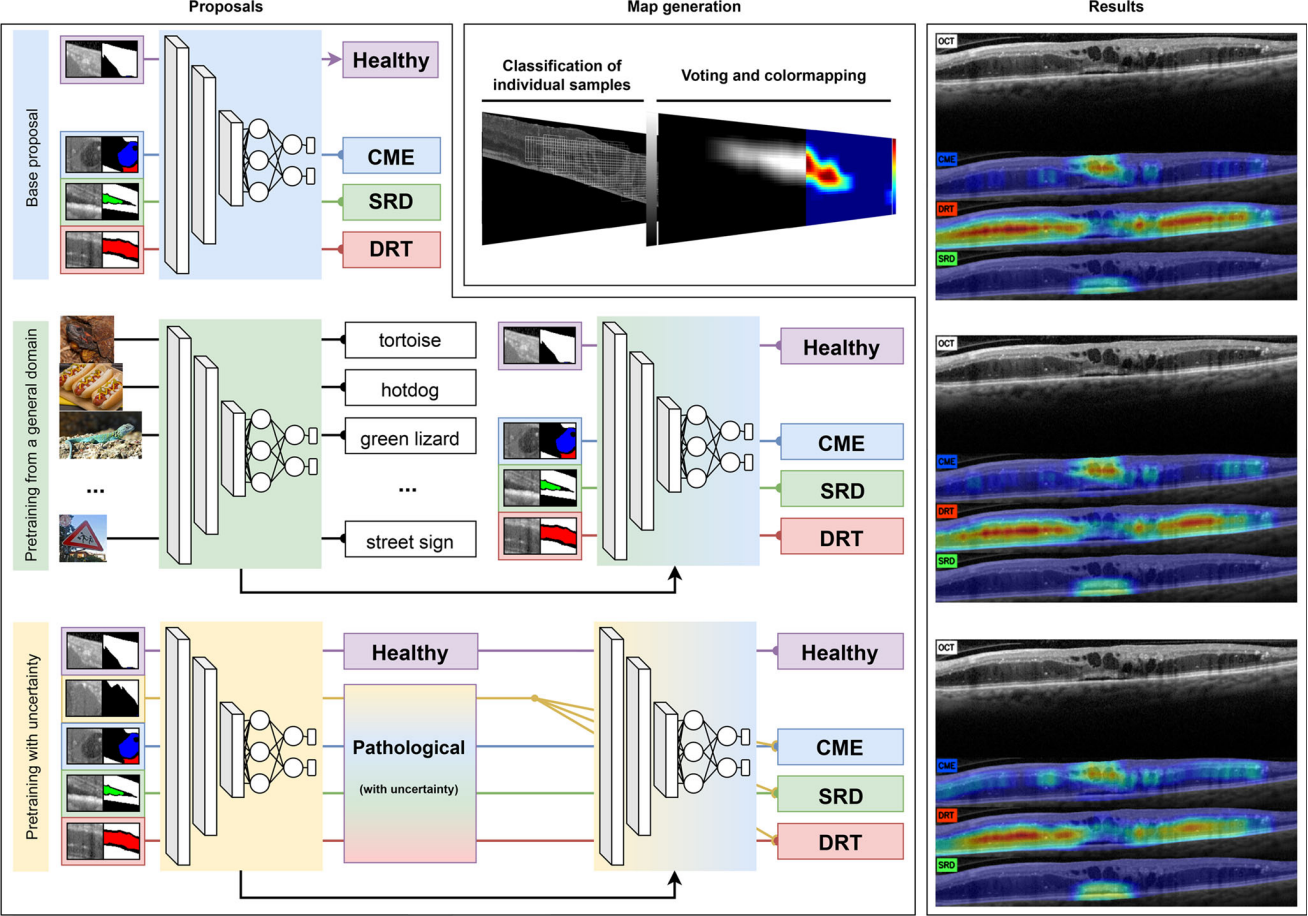
Diagram describing the three proposals and associated methodologies presented in this work

In this section, we explain each of the steps followed during the development of the proposed methodology (described in Fig. 4). In the first section, Section 3.1, we explain our three proposed approaches. Then, in Section 3.2, we describe the map generation strategy that is used in each approach to create the final representation of the DME fluid accumulations.

### Training of the models

For each of the experiment folds (explained in Section 2.1), three different models were trained using the configuration explained in Section 3.1.4. The first approach, presented in Section 3.1.1, shows the main proposal using a deep learning backbone. Then, in Sections 3.1.2 and 3.1.3, two additional proposals using this deep learning backbone but also taking advantage of transfer learning strategies.

#### First approach: a deep learning backbone

The first model to be trained is the one considered as baseline for our proposal. This model is trained from scratch using the samples extracted from the reference images and no prior knowledge is used. The four considered categories (Healthy, CME, DRT, and SRD) were established as target of the network. During this training, all the samples that were centered in a region labeled as uncertain were discarded, and only samples with a defined label are used.

#### Second approach: transfer learning from a general domain

As second approach, we use a network trained with the ImageNet dataset [32, 33]. This dataset consists of a set of images from the real world, such as from wildlife, food, and urban landscapes. This way, the dataset contains a total of 1000 target classes. This dataset is widely employed in the state of the art for works based on transfer learning thanks to its wide spectrum of included scenarios and large number of samples. The idea behind this knowledge transfer is that, due to its general purpose nature, the learned features allow for the resolution of a large number of problems requiring a minimal fine tuning to particular domains. Thus, the inherent uncertainty of our dataset could be supplied by features not contemplated in the baseline, as the features already learnt are adapted to our domain. This way, starting from the model trained with this wide spectrum dataset, we replace the classifier head of the network with the four classes considered in our target domain (keeping the rest of the network weights) and resume its training with the samples of each particular fold.

#### Third approach: transfer learning with uncertainty

As third approach, we first train a binary model to differentiate healthy from pathological samples. In this case, the healthy class corresponds to the same samples as in the previous scenarios, while the pathological class this time also includes the samples labeled as uncertainty in addition to the three clinical types of fluid accumulations (CME, DRT, and SRD). This forces the network to consider these regions, learning the filters and features associated with the uncertainty albeit not necessarily giving them a defined label. This way, by not assigning a defined class for it during the pretraining with uncertainty, the final network is aware of the patterns present in these mixed regions. Thus, in the next refinement stage, despite not explicitly being labeled and presented to the model, these patterns are taken into account when adjusting the gradients of the network.

Finally, after this initial pretraining in the domain, the fully connected classification layer of the network is replaced to one with the target DME subtypes: healthy, CME, DRT, and SRD types. We proceed with the knowledge transfer following the same configuration as in the other two scenarios: excluding the samples belonging to the uncertainty domain and exclusively using samples with a defined label by the experts. This approach is especially interesting for DRT, since it is mostly composed of vague patterns and their accumulations in the dataset are often labeled as uncertainty.

#### Training configuration

To train the four models, for the sake of repeatability and fair comparison, we employed the same configuration parameters. All the four networks were based on the DenseNet [35], as it demonstrated to be successful in works of similar domains [36, 37]. In particular, we used the DenseNet-161 configuration depicted in Table 1, where the convolutional layers were initialized using Kaiming initialization [38] and a random uniform distribution for linear layers. Additionally, before each *Transition Layer* and convolution in the *Dense Blocks*, a batch normalization and ReLU units are used. Before the classifier layer, the results are also batch-normalized. Each model was trained using a batch size of 250, as significantly smaller batch sizes stagnated without reaching convergence and were highly prone to overfitting. This value represented a good trade-off between training time and resource requirements, while higher batch sizes did not improve the results of the training. To further compensate for the dataset imbalance present (as some labels were more represented in the images than others), during the training, the samples were weighted proportionally to the number of remaining samples. For example, for the healthy class, the weight of a given sample is shown in Eq. 1.

1 $$  \mathrm{Healthy\ label\ weight}\ =\ \frac{\mathrm{CME\ +\ DRT\ +\ SRD}}{\mathrm{Healhty\ samples}} $$

**Table 1:** Basic structure of the DenseNet161-based network configuration used in this work

Layers	Convolution	Pooling	Dense block	Transition layer		Dense block	Dense block		Dense block	Transition layer		Dense block	Classification layer
Output size	32×32	16×16	16×16	16×16	8×8	8×8	8×8	4×4	4×4	4×4	2×2	2×2	−
*Structure*	7×7	3×3	$\begin {bmatrix} \!1 \times \!1 & \!conv \\ \!3 \times 3 & \!conv \end {bmatrix}$	1×1	2×2	$\begin {bmatrix} \!1\! \times \! 1 & \!conv \\ \!3 \!\times 3 & \!conv \end {bmatrix}$	1×1	2×2	$\begin {bmatrix} 1 \times 1 & conv \\ 3 \times 3 & conv \end {bmatrix}$	1×1	2×2	$\begin {bmatrix} 1 \times 1 & conv \\ 3 \times 3 & conv \end {bmatrix}$	Fully
	Convolution	Maxpool	× 6	Convol-	Average	× 12	Convol-	Average	× 36	Convol-	Average	× 24	connected
	Stride 2	Stride 2		ution	pool		ution	pool		ution	pool		softmax
					Stride 2			Stride 2			Stride 2		X classes

For the training of the binary model (healthy versus CME, DRT, SRD, and uncertainty class), this weight strategy is also used, but including this new label as shown in Eq. 2.

2 $$ \begin{array}{@{}rcl@{}} \mathrm{Healthy\ label\ weight} = \frac{\mathrm{Uncertainty \ +\ CME \ +\ DRT \ +\ SRD} }{\mathrm{Healhty\ samples }}\\ \end{array} $$

Additionally, the samples are randomly flipped horizontally with a probability of 50% to artificially increase the number of available samples in the dataset. This data augmentation strategy was chosen as the patterns present in the samples can appear in reality in both orientations.

As optimizer, we used the decoupled weight decay regularization Adam or AdamW [39] with AMSGrad stochastic optimization to improve convergence [40]. The initial learning rate and weight decay was set to 0.01 and the beta parameters as 0.9 and 0.999 with an epsilon of 1*e* − 08. A scheduler was implemented, so the learning rate was progressively reduced whenever the validation loss stagnated in factors of 0.66. This allows for smaller gradient steps the closer to the optimum valley the training got. The patience to reduce this learning rate was set to 10 epochs without validation loss improvement. Finally, the number of epochs of the training was also dynamically set, employing an early stopping strategy: the system would automatically stop if the validation loss did not improve for 25 epochs (which would allow for two learning rate scheduler steps and a margin of extra five epochs). The final model returned from the training is the one that obtained the best validation loss along all the epochs.

To evaluate the metrics, we use the area under the receiver operating characteristic curve (AUC), the F1 score, the accuracy, the precision and recall, and the Matthew’s correlation coefficient. The accuracy indicates the proportion of correctly classified samples, its formula being shown in Eq. 3 where TP are the true positives, TN the true negatives, FP the false positives, and FN the false negatives.

3 $$  \mathrm{Accuracy = \frac{TP + TN}{TP+TN+FP+FN}} $$

The Precision, in Eq. 4, evaluates the proportion of real positive samples from the total returned. On the other hand, the Recall, in Eq. 5, measures the proportion of real positive samples from the total in the dataset.

4 $$ \begin{array}{@{}rcl@{}} \text{Precision} &=& \frac{\text{TP}}{\mathrm{TP + FP}} \end{array} $$

5 $$ \begin{array}{@{}rcl@{}} \text{Recall} &=& \frac{\text{TP}}{\mathrm{TP+ FN}} \end{array} $$

The AUC (Eq. 6, where *x* represents a given score of the network from which a sample is considered positive) returns the probability of a given system of, when faced with a random positive sample, giving it a higher score than to another random negative sample.

6 $$  \text{AUC}={\int}_{x=0}^{1}\text{Precision}(\text{FPR}^{-1}(x))dx,\ \text{FPR}=1-\text{Recall} $$

Given this definition, to better understand its meaning, we can also define this AUC in terms of the Mann-Whitney-Wilcoxon test presented in Eq. 7, where *n* represents the positive-labeled scores and *m* the negative-labeled scores. This way, if the null hypothesis is rejected, we can infer that the values of the *n* distribution tend to exceed the *m* distribution (thus affirming its greater discriminative potential).

7 $$  \text{AUC} = \frac{  {\sum}^{n}_{i=1} {\sum}^{m}_{j=1} I(x_{i},y_{j})}{nm},\ I(a,b) = \left\{\begin{array}{cc} 1,\ \text{if}\ a > b \\ \frac{1}{2},\ \text{if}\ a = b \\ 0,\ \text{if}\ a < b \end{array}\right\} $$

The F1 score (Eq. 8) represents an alternative accuracy metric, being the harmonic mean of the Precision and the Recall. More robust to outliers and dataset imbalances than the traditional accuracy.

8 $$  \mathrm{F_{1}\ score}=\frac{2 \times \text{Precision} \times \text{Recall}}{\text{Precision} + \text{Recall}} $$

Finally, the Matthew’s correlation coefficient or MCC (Eq. 9) represents the correlation between the real labels versus the results returned by the methodology. In contrast with the other metrics, it ranges from − 1 to 1, where 0 MCC represents a random classifier, 1 MMC a perfect classifier, and − 1 MMC an inverse relationship between the real values and the ones returned by the classifier.

9 $$  \text{MCC} = \frac{\mathrm{TP \times TN - FP \times FN }}{\sqrt{\mathrm{(TP+FP)(TP+FN)(TN+FP)(TN+FN)}}} $$

Where possible, the metrics have been weighted as a per class basis to compensate for the imbalance present in the generated dataset.

### Confidence map generation

To analyze the behavior of the network, we only consider images where the network has not used even one window for training on a given fold. To generate said maps, we divide each retina into a series of overlapping samples of a given size (same as during training). After this extraction of overlapping samples, they are classified by the backbone convolutional neural network and assigned a label. Then, once all the samples have been labeled, a pixel-level voting is performed, where each pixel in the region of interest is assigned a confidence value. This value is defined as the proportion of windows that overlapped a given pixel that were classified belonging to a given class. Thus, a confidence of 80% in a CME region would indicate that 80% of the windows that had that pixel were classified as CME. Finally, since the system is intended for deployment in a real clinical environment, a cold-to-warm color mapping is applied with steep gradients. This will allow the expert to evaluate the nuances of the detections more easily to perform their diagnostic labor.

This way, in our work, these windows are uniformly acquired from the retinal region inside the OCT images with an overlap of 60px and a window size of 64px × 64px. This overlap represented a compromise between the number of total windows to be classified by the network and the quality of the final generated map. The number of windows that overlap a pixel is what determines the resolution of the final map confidence levels. Thus, lesser windows and the difference between the confidence levels in the map becomes steep, generating less robust maps (as fewer windows were used in the voting process, so misclassifications become more impactful in the final result). The same way, increasing the overlap between windows results into an exponential increase in resources needed for the generation of the maps.

## Results and discussion

We now proceed to analyze the results obtained for each experiment. First of all, in Section 4.1, we study the behavior and test metrics of the trained models for each approach (to consult the training and validation metrics, please refer to the Appendix in the Supplementary Material). In this first analysis of these results, we only take into account labeled samples towards the metrics, as the associated uncertainty values cannot be studied without a reference labeling. To fully study the behavior and performance of the approaches, we perform a fine-grained analysis of the final generated confidence maps in Section 4.2, where all the regions are taken into account to generate the final representation of the fluid regions.

### Training results

In this section, we present and analyze the final overall results of all three approaches. Then, we compare them with the state of the art and between themselves in Section 4.1.4.

#### Results of the baseline proposal with deep learning

The results of the model that was chosen following the early stopping strategy are shown in Table 2. While AUC indicates that the system consistently identifies positive samples with a higher value than negative samples, the MCC also confirms the strong positive relationship between most of the classes and their real value. Additionally, as established, DRT is the most complex case, obtaining lesser values in all the metrics compared to the other DME subtypes.

**Table 2:** Mean test and standard deviation of the cross-validation for the baseline proposal

	AUC	F1 score	Accuracy	Precision	Recall	MCC
Healthy	0.9744 ± 0.0043	93.92*%* ± 0.79	93.96*%* ± 0.78	94.01*%* ± 0.75	93.96*%* ± 0.78	0.8694 ± 0.0168
CME	0.9478 ± 0.0115	90.15*%* ± 1.16	90.19*%* ± 1.15	90.20*%* ± 1.14	90.19*%* ± 1.15	0.7652 ± 0.0262
DRT	0.9187 ± 0.0121	87.38*%* ± 1.11	87.19*%* ± 1.07	87.78*%* ± 0.91	87.19*%* ± 1.07	0.6760 ± 0.0264
SRD	0.9923 ± 0.0091	99.06*%* ± 0.41	99.06*%* ± 0.41	99.07*%* ± 0.41	99.06*%* ± 0.41	0.9361 ± 0.0275
Overall	0.9583 ± 0.0093	92.63*%* ± 0.87	92.60*%* ± 0.85	92.77*%* ± 0.80	92.60*%* ± 0.85	0.8117 ± 0.0832

#### Results of the transfer learning from a general domain

In Table 3, we present the test results of the cross-validation with transfer learning from the general domain. As we can see, overall, all the considered metrics have improved in comparison with the baseline proposal (and, as this baseline already surpassed the state of the art, this model surpasses it too). The transfer learning from the general domain has favored all points of view, improving all of them approximately the same percentage while slightly reducing their standard deviation (and, thus, indicating that the generated results are more robust than the baseline proposal).

**Table 3:** Mean test and standard deviation of the cross-validation for the proposal based on transfer learning from a general domain

	AUC	F1 score	Accuracy	Precision	Recall	MCC
Healthy	0.9757 ± 0.0047	94.08*%* ± 0.65	94.11*%* ± 0.64	94.15*%* ± 0.62	94.11*%* ± 0.64	0.8727 ± 0.0142
CME	0.9499 ± 0.0094	90.34*%* ± 0.96	90.40*%* ± 0.94	90.39*%* ± 0.96	90.40*%* ± 0.94	0.7693 ± 0.0229
DRT	0.9235 ± 0.0103	87.67*%* ± 0.90	87.48*%* ± 0.98	88.09*%* ± 0.77	87.48*%* ± 0.98	0.6840 ± 0.0225
SRD	0.9922 ± 0.0103	99.10*%* ± 0.45	99.10*%* ± 0.45	99.11*%* ± 0.45	99.10*%* ± 0.45	0.9402 ± 0.0280
Overall	0.9603 ± 0.0087	92.80*%* ± 0.74	92.77*%* ± 0.753	92.94*%* ± 0.70	92.77*%* ± 0.75	0.8166 ± 0.0219

#### Results of the transfer learning with uncertainty

As shown in Table 4, the model that was trained taking advantage from regions with inherent uncertainty is able to attain comparable performance to the model pretrained from the general domain, but only requiring a reduced set of images. However, as mentioned, these test metrics only consider labeled samples, not taking into account the samples with uncertainty. Thus, while we can assess that the behavior of this model is comparable to the model pretrained with ImageNet in labeled regions, we further study the advantages of this pretraining with uncertainty in the fine-grained analysis of the generated maps in Section 4.2.

**Table 4:** Mean test and standard deviation of the cross-validation for the proposal based on transfer learning with uncertainty

	AUC	F1 score	Accuracy	Precision	Recall	MCC
Healthy	0.9742 ± 0.0043	94.20*%* ± 0.74	94.24*%* ± 0.72	94.29*%* ± 0.68	94.24*%* ± 0.72	0.8755 ± 0.0157
CME	0.9553 ± 0.0084	90.39*%* ± 0.74	90.47*%* ± 1.00	90.44*%* ± 1.00	90.47*%* ± 1.00	0.7704 ± 0.0238
DRT	0.9264 ± 0.0083	87.51*%* ± 0.82	87.27*%* ± 0.89	88.04*%* ± 0.71	87.27*%* ± 0.89	0.6821 ± 0.0191
SRD	0.9915 ± 0.0083	99.05*%* ± 0.47	99.04*%* ± 0.48	99.07*%* ± 0.44	99.04*%* ± 0.48	0.9368 ± 0.0294
Overall	0.9619 ± 0.0073	92.79*%* ± 0.69	91.76*%* ± 0.77	92.96*%* ± 0.71	92.76*%* ± 0.77	0.8162 ± 0.0220

#### Performance comparison with previous works and between approaches

In Table 5, we present the results comparing our three proposals with the state of the art. To the best of our knowledge, the only work that addressed the issue of the characterization of DME with this diffuse paradigm is Vidal et al. [30]. As shown, our proposals based on a deep learning backbone surpass the performance reported in said work. Moreover, we see how both approaches based on transfer learning are able to reach similar performance. Additionally, the approach pretrained with uncertainty required a significantly lesser number of samples.

**Table 5:** Accuracy results of all three proposals and most recent work of the literature for each of the DME categories

	Vidal et al. [30]	Deep baseline	General domain	From uncertainty
CME	84.04*%* ± 3.67	90.19*%* ± 1.15	90.40*%* ± 0.94	90.47*%* ± 1.00
DRT	78.94*%* ± 3.64	87.19*%* ± 1.07	87.48*%* ± 0.98	87.27*%* ± 0.89
SRD	95.40*%* ± 2.02	99.06*%* ± 0.41	99.10*%* ± 0.45	99.04*%* ± 0.48

While the approaches based on knowledge transfer seem to attain a slight improvement over our baseline proposal, in the following fine-grained analysis of the generated confidence maps with each of the models, we further study their differences.

### Test map analysis

**Fig. 5:**
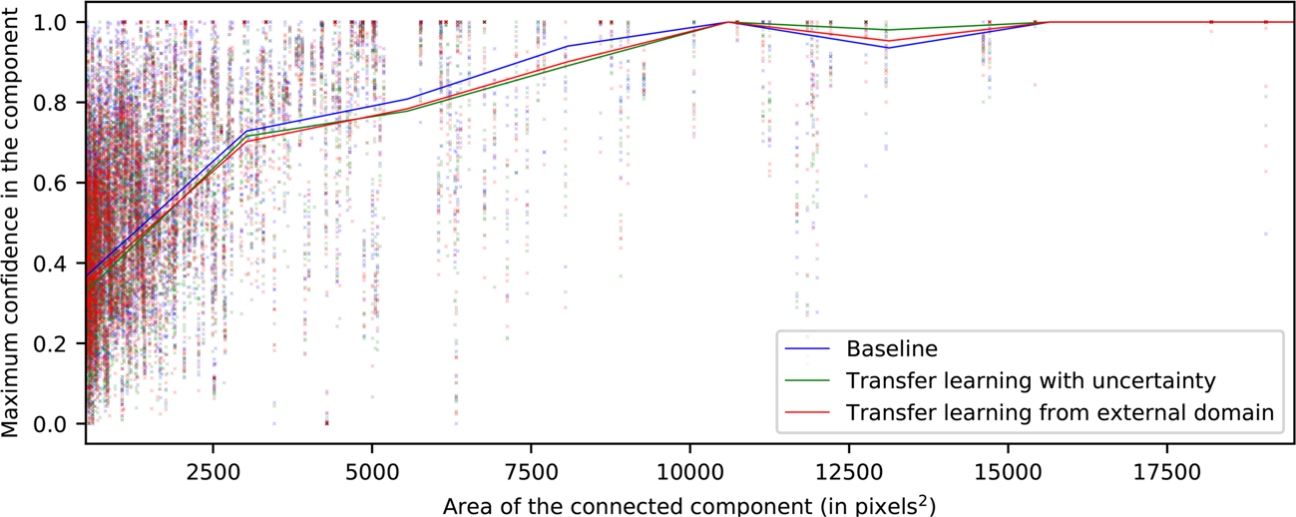
Maximum confidence attained inside each labeled connected component for the CME class

**Fig. 6:**
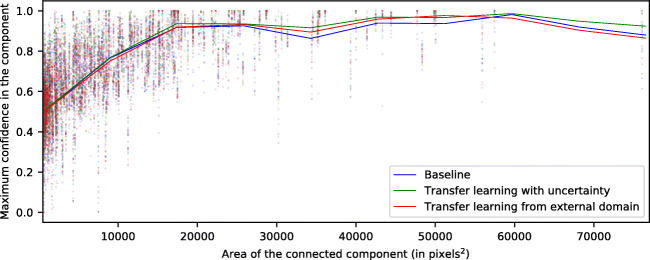
Maximum confidence attained inside each labeled connected component for the DRT class

**Fig. 7:**
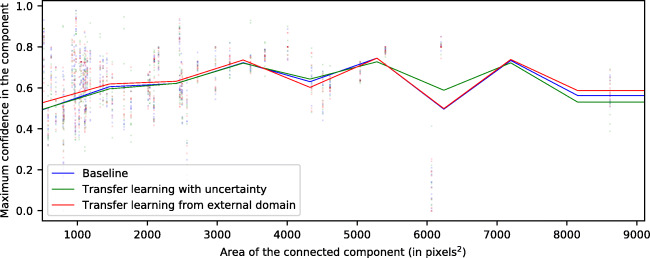
Maximum confidence attained inside each labeled connected component for the SRD class

Below, in Figs. 5, 6, and 7, we present an analysis of the confidence from the test maps. In said figures, we examine each of the connected components of the reference labeling and study the maximum confidence assigned to it by generated the map. In each of the figures, for each type of pathology, we show a point cloud for each maximum confidence assigned to each connected component according to its size. Additionally, we include a trend line to facilitate its visualization. This trend line has been calculated using a sliding window strategy based on the median value and covering a range of 1000 pixels of connected component sizes, advancing the window by 10 units in 10 units. This sliding window has been smoothed by an interpolation by b-splines of degree 3 and 10 points of resolution.

First of all, in Fig. 5, we note a logarithmic trend between the relationship of the connected component size and the maximum confidence assigned to it for the CME class. Thus, we can infer that the system tends to assign lower confidence to microcystic fluid accumulations. On the other hand, in Fig. 6, the models are shown to be more stable for the DRT class. We also see a higher confidence per connected component as its size increases, the same way as with CME. However, overall, the results show a slightly lower maximum confidence for the DRT class.

In Fig. 7, we present the analysis for the third type of DME fluid accumulation: SRD. Unlike in the previously mentioned cases, in this scenario, we do not see the logarithmic relationship between size and performance of the model. Thanks to the morphological consistency of this type of fluid accumulation, the model does not depend on texture and intensity constraints alone for its classification.

This type of fluid accumulation usually appears in a region where the retinal layers exhibit very characteristic patterns and, at the same time, the fusiform deformation is very recurrent in the vast majority of instances. Because of these factors, the associated confidence is able to remain largely stable regardless of its size. In the other two types of DME (CME and DRT), the irregular shapes that the accumulations may present negatively affects their detection, depending almost exclusively on texture and intensity features (which, as stated, often are intermingled between classes). Additionally, these patterns are especially sensitive to the device capture conditions, which can affect brightness, contrast, and even device noise in the generated OCT image. Finally, SRD presents a mean confidence around 60–70%, below the metrics that are obtained in the other cases. This is due to the reduced number of samples available for this particular pathology compared to the other two cases, which possibly decreases its weight during training (despite the established data augmentation and weighting strategies to compensate this phenomenon).

**Fig. 8:**
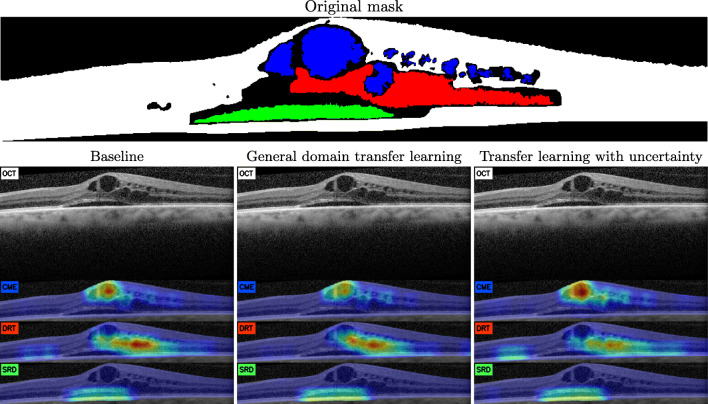
Example of generated maps from the Spectralis device with the three considered types of fluid accumulations

Below, we present different cases of test maps generated by each of the proposals to illustrate the aforementioned scenarios, as well as a commentary relating them to scenarios seen in this previous analysis. First of all, as we can see in Fig. 8, the three models find all the fluid accumulations established in the reference labeling. However, as shown in the previous analysis of connected components, the confidence levels are lower in the smaller fluid accumulations. It is in the particular case of the model pretrained with uncertainty that this confidence is more homogeneously preserved within the region indicated as pathological.

We can speculate that, given the maps are generated based on the overlapping of windows, in these smaller fluid accumulations the maps present an inherent lesser maximum confidence value. A sampling density based on extraction by connected component rather than a fixed density would help to compensate for the sampling of this subtype of DME.

In that same image, we can also see the effect generated in the SRD, even in the rare scenario where its extent is considerable. The maximum confidence associated with this type of DME is lower than in other cases. However, the confidence along the connected component is very homogeneous. Thus, despite the lower maximum confidence, the methodology still offers robustness and repeatability for the analysis by clinical experts, successfully integrating information from numerous independent windows and domains for the generation of the current representation.

**Fig. 9:**
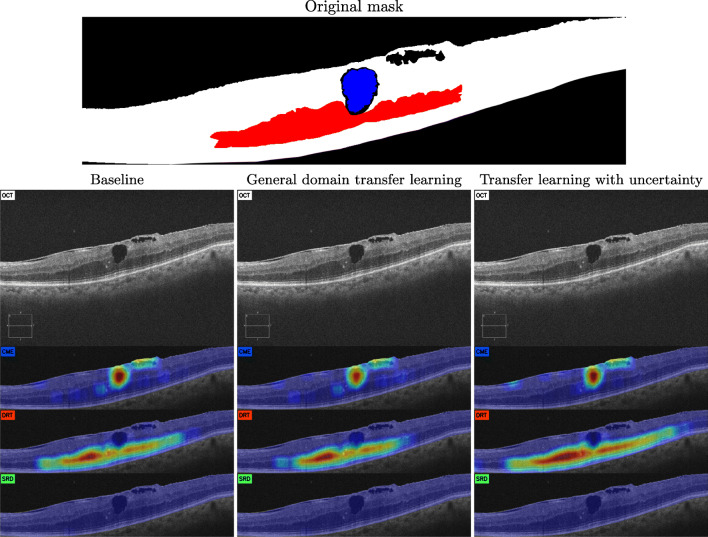
Example of generated maps from the Cirrus device with CME and DRT fluid accumulations

In Fig. 9, we see an example where DRT, the most complex type of fluid accumulation (and usually associated with uncertainty) is the one that benefited the most by the approach based on transfer learning from uncertainty. Not only the extent of the region indicated as DRT is also significantly larger than in the other two proposals, it also presents higher overall confidence. This improvement is especially explicit in the images coming from the Cirrus device, since the processing this device performs on the images tends to be detrimental to the DRT texture and intensity patterns.

**Fig. 10:**
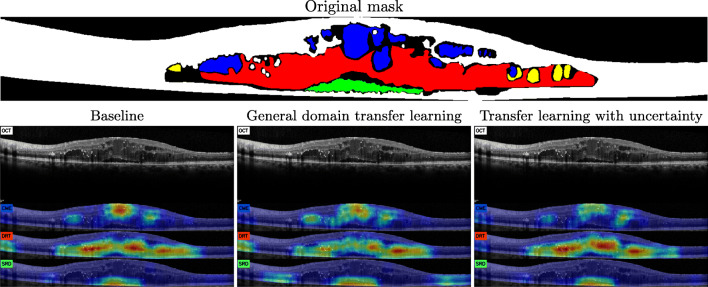
Example of generated maps from the Spectralis device with an advanced stage of all three types of fluid accumulations. Yellow represents lipidic fluid accumulations, in this work considered as belonging to regions with uncertainty

This same phenomenon can be seen in Fig. 10, a quite complex scenario where the class with DRT is better represented in the uncertainty-based model. Moreover, it can be seen how the training including samples from the regions labeled as uncertain has prevented it from incorrectly classifying the dark regions caused by dense bodies to the left of the retina (as happened in both the model trained from scratch and the transfer learning from an external domain). Similarly, the transfer learning from the external domain also returned additional false positives in the SRD type. These detections are overlapping small retinal deformations (probably caused by an incipient DRT) that adopt an slight dome-like shape. This indicates that this model has favored the morphological descriptor over the indicators from the texture features from the outermost layers of the retina. Albeit this strategy returns satisfactory results as well as the other two models in the correct regions, we see how the patterns derived from a generalist domain make the model more prone to these false positives.

**Fig. 11:**
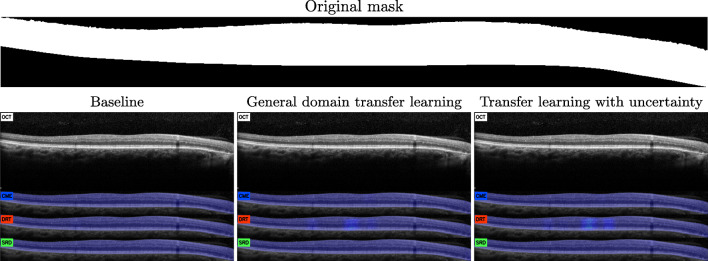
Example of generated maps from the Spectralis device without fluid accumulations

Finally, in Fig. 11, we present an illustrative case where no fluid accumulations are present. All models (and especially the model with uncertainty) have returned a negligible confidence in a healthy region for the DRT category. This phenomenon is possibly a consequence of the previous cases, in which the model trained with uncertainty favors the DRT class. It is possible that the same thing that gives it an advantage when detecting more complex cases, also has the trade-off of negligible false positive responses when analyzing completely healthy scenarios. However, we see that none of the approaches wrongly detected the shadow caused by a vessel passing through the retina.

Overall, the experiments demonstrate that all the approaches outperform the state of the art and how the transfer learning approaches help to improve the behavior of the system regardless of the device with which the images were taken. In addition, the approach that takes advantage of the inherent uncertainty of the system is able to obtain similar results than with transfer learning from a domain with thousands of images, but needing significantly less samples and resources. However, this approach has also demonstrated to outperform the other two in regions with remarkable complexity and uncertainty. Finally, this approach also suffered the least in regions with microcysts (although obtaining dim detections nonetheless). Thus, all three approaches have demonstrated their suitability for helping clinicians to detect and classify the diffuse regions considered in the domain (that would, otherwise, be subject to the subjectivity of the clinician).

## Conclusions

In this work, we present three approaches for the detection and characterization of the three types of DME in retinal OCT images, one of the main causes of blindness in developed countries. Due to the diffuse nature of these accumulations, in the literature, a specific paradigm has been developed to address their detection and characterization. However, until now, said paradigm was only contemplated with classical learning strategies. Furthermore, the information of the regions with uncertainty was not explicitly considered in such works and, usually, the inference on these regions was left to the criterion of the intelligent system.

Thus, in this work, we have presented the first work capable of characterizing the three types of DME on this diffuse paradigm using a deep learning backbone. Additionally, we addressed the problem of associated uncertainty by means of two other approaches based on transfer learning. One of them by means of a knowledge transfer from a general domain and other from the same domain taking advantage of patterns usually lost in regions with an undefined label.

The results of our three proposals are highly satisfactory. The approach with a backbone based on deep learning has proven to far surpass the state of the art based on classical learning methodologies. The same way, the approaches that take advantage of transfer learning strategies have outperformed this baseline in particular complex scenarios such as the difficult DRT. Moreover, the performance obtained with our approach pretrained in the same domain taking advantage of the uncertainty is able to achieve similar results to the pretrained model in a generalist domain with significantly fewer images. Finally, the fine-grained study on the performance of the generated confidence maps shows that this approach, while obtaining similar results overall, shows a more robust behavior in boundary regions with associated uncertainty.

As future work, we plan to address the particular challenge of microcysts, a subset of cystic bodies with their own unique properties. This subset of cystic bodies seems to significantly increase the sparsity of the models, suggesting that their features should be considered as a distinct class (as is done in the clinical domain) rather than as part of the CME subtype of DME. In the same way, it would be interesting to study a mixed approach between the generalist domain pretraining and the one that considers uncertainty, to address their weaknesses and complement their strengths. Finally, it would be interesting to adapt our proposals to other pathologies and medical imaging domains with similar diffuse features and associated uncertainty.

## Electronic supplementary material

Below is the link to the electronic supplementary material.

(PDF 233 KB)
