# Supplementary material for: Multivendor fully automatic uncertainty management approaches for the intuitive representation of DME fluid accumulations in OCT images
Source: Med Biol Eng Comput. 2023 Jan 24;61(5):1209–24. doi: 10.1007/s11517-022-02765-z (PMC10083163; doi:10.1007/s11517-022-02765-z)
Supplement: Supplementary file 1 — (PDF 233 KB) [file 11517_2022_2765_MOESM1_ESM.pdf]

Appendix

Below, we present the training results per epoch for each of the proposals. In this section, we analyze the behavior and progression throughout all the training epochs.

Training of the baseline.

In Figure 1, we present the training results for the baseline proposal: a model trained from scratch without any kind of transfer learning. In all the contemplated types of DME a satisfactory AUC was achieved, with optimal stability towards the end of the training process. Moreover, since throughout all the epochs the validation and test metrics are extremely similar, we can affirm that the used dataset contains sufficient representativeness to be used for this particular study.

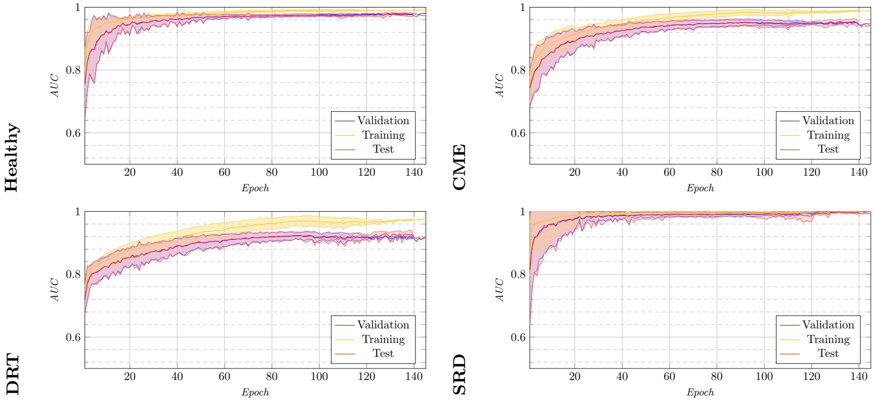

Fig. 1 Average AUC and its standard deviation during training for each considered class for the baseline model.

These graphs reflect the inherent difficulty of each of the DME types, especially in the case of DRT with a lower curve slope. On the other hand, both SRD and Healthy classes show how scenarios with high intra-class homogeneity allow for less sparsity to be taken into account with the model, resulting in a more stable standard deviation.

Training of the transfer learning from a general domain.

Next, in Figure 2 we present the results for the model pretrained with the ImageNet dataset. While the progression (as expected) follows the same behavior of the baseline, the overall sparsity of all classes has sensibly diminished.

We specially see a noticeable improvement during training for SRD, which obtains significantly better results in its initial epochs. This suggests that, indeed, the transfer learning from the external domain has helped, as the filters needed for this subtype of DME were learnt earlier in spite of the smaller

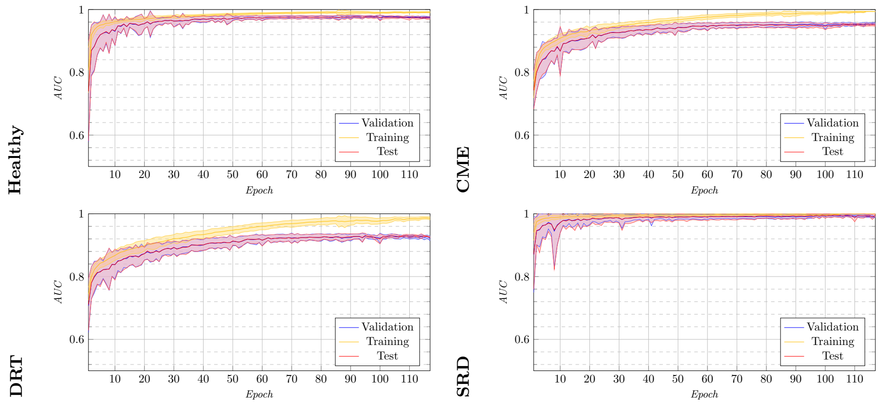

Fig. 2 Average AUC and its standard deviation during training for each considered class for transfer learning from ImageNet (external domain).

number of samples. However, since the final epochs attain similar performance, we can therefore assume that this transfer learning from the external domain would mostly serve to accelerate the training procedure. As shown, the model reaches stability sooner than the our proposal trained from scratch. Nonetheless, as mentioned, these graphs only study performance on regions with a known label, and the fine-grained study of the behavior in regions with associated uncertainty revealed improvements over the baseline.

Training of the transfer learning with uncertainty.

Finally, in Figure 3, we present the training behavior of the model pretrained with the same domain but including information of the regions with associated uncertainty. In this figure, evidence of the results of the knowledge transfer from the same domain can be seen: a refinement of the filters already learnt towards a more fine-grained classification that considers all four subtypes. All categories quickly reach stability after a few epochs, as all the filters needed were learnt during the binary pretraining stage and only minor finetuning is required. This way, the number of epochs that are needed to achieve the completion of the training is comparable to the number needed for the model that received a knowledge transfer from the external domain (without evidence of overfitting). Nonetheless, thanks to the early-stopping strategy, we can also ensure that the uncertainty-related filters are preserved (shown in the results of the generated maps fine-grained analysis).

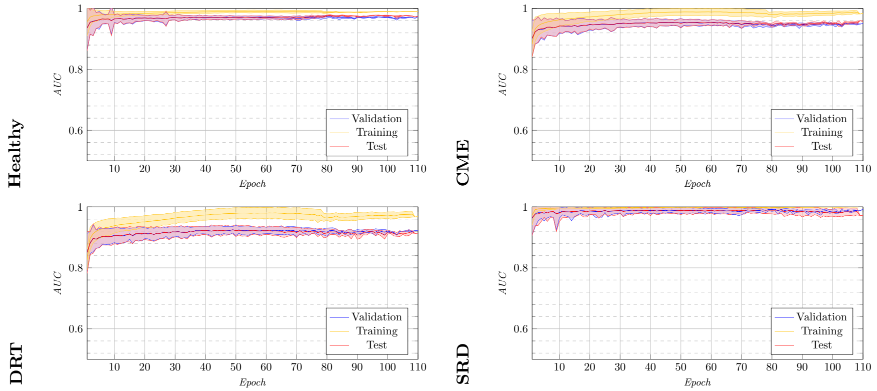

Fig. 3 Average AUC and its standard deviation during training for each considered class for the model with transfer learning from uncertainty.
